# ^13^C-NMR Spectroscopy and Elemental Composition of Humic Acids of Brown Forest Soils and Sod-Brownzems of the Southern Vitim Plateau (Russia, Baikal Region)

**DOI:** 10.3390/molecules31040606

**Published:** 2026-02-09

**Authors:** Erzhena Chimitdorzhieva, Tsypilma Korsunova, Yurii Tsybenov, Nimbu Baldanov, Elena Valova

**Affiliations:** 1 Institute of General and Experimental Biology, Siberian Branch, Russian Academy of Sciences, Sakhyanovoy St., 6, 670047 Ulan-Ude, Russia; 2 Institute of Land Management, Cadastre and Land Reclamation, Buryat State Agricultural Academy Named After V.R. Filippova, Pushkina St., 8, 670034 Ulan-Ude, Russia; 3 Institute of Natural Sciences, Buryat State University, Smolina St., 24 a, 670000 Ulan-Ude, Russia

**Keywords:** carbon, ^13^C-NMR spectroscopy, elemental composition, humus pockets, humic acids, brown forest soils, sod-brownzems, Vitim plateau

## Abstract

This study shows that the structural features of humic acids reflect the specific characteristics of organic matter in permafrost soils of the southern Vitim Plateau. The region’s extracontinental climate determines the rate of decomposition, the depth of humification, and the chemical structure of humic acids. Brown forest soils (Haplic Cambisols) and sod-brownzems (Leptic Cambisols Skeletic) contain high amounts of organic carbon and total nitrogen in their upper horizons but differ in their vertical distribution. Brown forest soils are characterized by a sharp decrease in organic carbon content with depth and the presence of humus pockets enriched in carbon and exchangeable bases. Sod-brownzems contain more organic carbon with increase in acidity and base loss with depth. Both soil types retain satisfactory natural fertility. ^13^C nuclear magnetic resonance spectroscopy data reveal marked differences in the structural maturity of humic acids. Humic acids from the A horizons of brown forest soils contain an equilibrium combination of aliphatic and aromatic structures, a well-developed system of oxygen-containing groups, and moderate condensation, indicating an intermediate stage of humification. Humic acids from humus pockets are more aromatic and highly humified. They reflect an advanced stage of humification and possess high chemical stability. Humic acids from sod-brownzems also exhibit high aromaticity, which facilitates the formation of stable organomineral complexes. A comparison of the samples reveals a consistent increase in aromaticity, condensation, and stability from the A horizons of brown forest soils to the A horizons of sod-brownzems and further to humus pockets. This progression corresponds to an increase in humification and a decrease in the mobility and bioavailability of organic matter. These results confirm that the structural characteristics of humic acids are determined by soil type and formation conditions. Elemental composition revealed that humic acids from brown forest soils are characterized by the highest aromaticity and maturity, while humic acids from HA-brown forest soils-A have a less condensed structure. Humic acids from sod-brownzems occupy an intermediate position, combining high aromatization with a moderate degree of humification. Overall, the obtained elemental composition data are fully consistent with the results of ^13^C NMR spectroscopy, mutually confirming the identified structural features and the degree of transformation of soil organic matter.

## 1. Introduction

Humic acids (HA) are a key component of soil organic matter (SOM), forming a stable, difficult-to-decompose fraction that determines the structure, fertility, and ecological balance of the soil system. Nuclear magnetic resonance (NMR) spectroscopy is widely used to study the chemical structure of HAs, allowing the identification of molecular features of their composition [1]. Determining the elemental composition of soils is also an important analysis, providing insight into the chemical structure of HAs and their role in soil biogeochemical processes [2].

^13^C nuclear magnetic resonance (^13^C-NMR) is a powerful tool for molecular analysis of SOM, allowing the separation of carbon structures by the type of functional groups (aliphatic, O-substituted, aromatic, or carboxyl) and quantitative assessment of the degree of aromatization and functionalization of organic material [1]. There are many studies in the literature on the molecular structure of humic substances obtained by ^13^C NMR [3,4,5,6,7]. Current understanding of the role of HAs emphasizes that their molecular structure is directly linked to key ecological functions of soils, including carbon sequestration, resistance to decomposition, moisture retention, control of metal mobility, and protection of organic compounds from rapid mineralization. Changes in the ratio of aliphatic, O-substituted, and aromatic structures determine the stability of humic complexes and the rate of carbon cycling in the ecosystem [1]. For cold-zone regions, where organic matter decomposition is limited by temperature, the structural features of HAs are crucial for long-term carbon accumulation.

Modern literature shows the high efficiency of the ^13^C-NMR method for assessing the molecular structure of humic substances in natural and agrogenic soils. For example, Baldock and Preston [8] showed that solid-state ^13^C-NMR spectroscopy allows for the reliable separation of aliphatic, O-alkyl, and aromatic fragments, determining the degree of organic matter decomposition. Rumpel and Kögel-Knabner [9] used this method to determine the proportion of stable, microstructurally protected carbon in forest soils. The work of Simpson et al. [10] presented direct evidence of changes in humus structural groups during degradation and mineralization. Thus, extensive experimental data demonstrate the reproducibility and validity of ^13^C-NMR as a key method for the molecular analysis of SOM [7]. To date, ^13^C-NMR spectroscopy remains one of the most informative methods for studying the structure of HAs, allowing the determination of the ratio of aliphatic, aromatic, carboxyl, and carbonyl fragments.

Additionally, one of the most important and stable identification characteristics of HAs is their elemental composition [2], which provides information on the general principles of molecular structure, the level of condensation, and the degree of humification. Modern studies show that the elemental composition of soil HAs reflects the degree of organic matter transformation and serves as an indicator of soil biogeochemical processes [11,12]. HAs are characterized by the predominance of carbon and oxygen, while the content of hydrogen, nitrogen, and sulfur varies depending on the soil type, humification conditions, and the degree of aromatization [13]. In recent years, the importance of elemental composition analysis has been emphasized not only for the characterization of SOM but also for assessing the resilience of ecosystems under anthropogenic impact [13].

Boreal forest soils function as a terrestrial network sink in the global carbon cycle [14]. Russia’s boreal forests are among the largest continuous forests on Earth, covering 20% of the world’s forest area and 70% of the world’s boreal forest area [15]. Boreal forest productivity has changed following climate warming [16], and there is potential for carbon sequestration in boreal forests that could change in the next century due to climate change [17]. The direction in which soil carbon stocks change will depend on how SOM stabilization mechanisms respond to warming, increased nitrogen input, and the amount of plant residues in the soil [18]. In this regard, studies of stabilized organic matter in Russian forest soils are of great interest for the molecular understanding of the storage forms of carbon compounds [19,20,21]. Despite the large number of studies devoted to the ^13^C-NMR and elemental composition characteristics of HAs, data on HAs in permafrost-affected forest soils remain extremely limited.

The Baikal region is sensitive to global warming, and any changes in organic matter stabilization in forest soils can significantly impact the regional carbon balance. Therefore, ^13^C-NMR analysis and elemental composition of humic acid allows us to identify molecular signatures of potential weakening or strengthening of soil carbon sequestration capacity under climate change. This study makes an important contribution to understanding the response of cold soils to climate trends and to assessing the risks of soil decarbonization in the Baikal region. An analysis of the available literature reveals that studies on the structure of HAs in brown forest soils and sod-brownzems of the Vitim Plateau are virtually nonexistent. Existing studies of the soils we studied are limited primarily to the morphological and physical characteristics of the soil profiles. ^13^C-NMR spectroscopy data and the elemental composition of HAs are also available for other soil types in Western Transbaikalia—meadow chernozem, chestnut soil, and chernozem.

In this context, regional studies are particularly important, allowing us to identify the spatial variability of organic carbon accumulation and stabilization processes under various environmental conditions. Data from different climatic regions are necessary for a full understanding of the mechanisms regulating various input sources [9].

In the Russian soil classification, brown forest soils are classified as burozems and are characterized by a poorly differentiated profile with a Bm horizon, corresponding to the Haplic Cambisols type in the WRB classification. Sod-brownzems, formed through the sod process, are distinguished as a separate subtype with a distinct humus A horizon and a thin profile on coarse-grained rocks; in the WRB system, they correspond to Leptic Cambisols Skeletic.

The aim of this study is to analyze and interpret the ^13^C-NMR spectra and elemental composition of HAs in brown forest soils and sod-brownzems in the southern Vitim Plateau.

The scientific and practical significance of this work lies in the fact that the obtained molecular data will help clarify the mechanisms of OM formation and stabilization in soils in the southern Vitim Plateau, provide a basis for regional assessments of the carbon pool and its potential response to climate change, and also be useful in developing measures for land management and soil carbon conservation.

^13^C-NMR analysis and elemental composition of HAs has significant applied and research potential. The resulting parameters allow them to be used as indicators of the degree of humification, stability of organic carbon, and soil-forming conditions, which is important for monitoring the condition of brown forest and sod-brownzems in cold regions. The identified structural fragments and elemental composition of organic matter enable more accurate fertility predictions and assessment of the risks of carbon loss due to climate change. Furthermore, the study results provide a new factual basis for interregional comparisons and refinement of carbon cycle models, thereby significantly expanding our understanding of humus formation and transformation in Eastern Siberia.

## 2. Results

### 2.1. Forest Vegetation

On brown forest soils, a birch–aspen–larch forest with mixed forbs and lingonberry undergrowth grows. *Larix gmelinii* (Rupr.) Rupr forms the tree layer. *Populus tremula* L. and *Betula platyphylla* Sukaczev are also present in the stand. The stand has average characteristics: quality classes 2–4, crown density of 0.4–0.6, projective crown cover of 40–50%, height of 15–22 m, average trunk diameter of 16–31 cm, and uneven trunk density. The undergrowth is sparse, uneven in the windows, with an average height of 1.5–1.8 m. It is represented by *Rhododendron dauricum* L., with a total projective cover (TPC) of 5–10%. The undergrowth also includes *Cotoneaster melanocarpus* Fischer ex Blytt and Rosa acicularis Lindley. The herbaceous–dwarf shrub layer is low, uneven in density and height. TPC is 70%, in places up to 35%. The average height of the layer is 20 cm and the maximum is 40 cm. *Vaccinium vitis-idaea* L. predominates and *Maianthemum bifolium* (L.) F.W. Schmidt and Pyrola asarifolia Michaux are abundant. *Galium boreale* L., *Calamogrostis* sp., and *Carex pediformis* C.A. are also present, as are Meyer, *Pulsatilla flavescens* (Zucc.) Juz., *Vicia unijuga* A. Br., *Lathyrus humilis* (Ser.) Sprengel, *Sanguisorba officinalis* L., etc. Moss–lichen cover is not developed.

On sod-brownzems, the tree layer is predominantly represented by *Larix gmelinii*, with *Betula dahurica* Pall. among the associated species. The tree stand is characterized by sparseness and small trees: height 15–20 m, average trunk diameter 15–17 cm. Crown density varies between 0.3–0.5, with total projective crown cover of approximately 30–45%. The understory is sparse and unevenly distributed. It is dominated by *Rhododendron dauricum* L. with a total projective cover of 5–15%. The herbaceous–dwarf shrub layer is characterized by uniformity and limited species composition. It is primarily composed of *Calamagrostis* sp., *Carex* sp., *Sanguisorba officinalis* L., *Geranium* sp., and *Galium* sp. The projective cover of the herbaceous layer reaches 60–70%, with an average height of 20–40 cm. The height and density of the herbaceous layer are uneven, with patches of sparse grass growth. The moss–lichen cover is unevenly distributed and common, but rarely forms a continuous carpet; sphagnum mosses and leafy-stemmed species are present.

### 2.2. Physico-Chemical Characteristics of Soils

Brown forest soils (Haplic Cambisols). In brown forest soils, the organic carbon (Corg) content in the 0–10 cm soil layer is 4.9 ± 0.65%, and in the 20–30 cm layer this value decreases sharply to 0.77 ± 0.09% (Table 1). In the soil material of the humus pocket at a depth of 10–20 cm, the carbon content is high 5.1 ± 0.76%, and in the 20–30 cm layer Corg is 3.7 ± 0.92%, which indicates carbon immobilization in the cryogenic reservoir. Average enrichment of the soil with nitrogen was revealed; its amount varies from 0.21 ± 0.01 to 0.63 ± 0.02% in the soil profile and from 0.35 ± 0.03 to 0.64 ± 0.02% in the soil material of pockets. The AY horizon and the humus pocket soils exhibit high exchangeable Ca and Mg—35.9–36.6 cmol(eq)/kg. The soil has a slightly acidic pH.

In the soil profile, complex cryogenic cracking processes complicate the morphological structure, primarily resulting in variations in the Corg content across the soil profile. The coefficient of variation for Corg content is 15–30% in the soil profile and 29–50% in the pockets.

The studied brown forest soils are medium loamy in texture, with a slight increase in weight further down the profile. The physical clay content in the humus pocket soils is relatively uniform and does not exceed 38.75%.

Sod-brownzems (Leptic Cambisols Skeletic). In the top 0–10 cm soil layer, the Corg content is 6.03 ± 0.40% (Table 1), reflecting a high saturation with organic matter due to the slow mineralization of plant residues in a cold and humid climate. Total nitrogen reaches 0.59 ± 0.05%, indicating active humus accumulation and moderate biological productivity. The soil reaction is slightly acidic, pH = 5.70 ± 0.05. Exchangeable Ca and Mg are 31.50 ± 0.34 cmol (eq)/kg, indicating a good supply of calcium and magnesium cations. In the 10–20 cm layer, a sharp decrease in the carbon content to 2.03 ± 0.20% and nitrogen to 0.25 ± 0.02% is observed, which is associated with a weakening of the influence of plant residues and the transition to the mineral part of the profile. Acidity increases pH = 5.20 ± 0.05, exchangeable Ca and Mg decreases to 14.90 ± 0.31 cmol (eq)/kg, reflecting a decrease in buffering capacity. In the lower layer (20–30 cm), the proportion of Corg is minimal at 0.50 ± 0.05%, and nitrogen decreases to 0.20 ± 0.01%. The reaction of the medium is most acidic at pH 5.10 ± 0.04, and the amount of exchangeable Ca and Mg drops to 6.90 ± 0.45 cmol (eq)/kg.

In general, sod-brownzems are characterized by a high organic matter content in the upper layers, a significant gradient of carbon and nitrogen, increased acidity with depth, and a gradual decrease in the amount of exchangeable bases. The profile is characterized by a gradual decrease in clay content and an increase in coarse sand content to 60–80% in the lower horizons. This structure promotes good drainage and aeration.

### 2.3. ^13^C-NMR Spectra of HA

Integral values of signal intensity by spectral regions are presented in Table 2.

Description of spectral ranges (Table 2).

0–48 ppm. This zone is characterized by signals of aliphatic carbon atoms (CH_3_, CH_2_). HA-brown forest soils-A and HA-sod-brownzems-A have high and similar values (17–19%), while HA-brown forest-pocket is lower (11.6%). The high contribution indicates the predominance of aliphatic chains and side groups in the HA-brown forest soils-A and HA-sod-brownzems-A. 

48–90 ppm. This range belongs to aliphatic C atoms linked to heteroatoms (O, N)—fragments of carbohydrates, C–O, and C–N bonds. HA-brown forest soils-A (17.7%) is higher than HA-brown forest-pocket (11.2%) and HA-sod-brownzems-A (12.5%). Higher values indicate a higher number of functionalized aliphatic units in HA-brown forest soils-A. 

90–108 ppm. This range corresponds to acetal and conjugated carbons of polysaccharides and vinyl structures. The contribution is small in all samples. 

108–145 ppm. This range is attributed to aromatic and condensed aromatic carbons. The HA-brown forest-pocket sample clearly has the highest aromaticity, followed by HA-sod-brownzems-A, and then HA-brown forest soils-A—this is the key structural distinguishing feature. 

145–167 ppm. This range characterizes O-substituted aromatic carbons. HA-brown forest soils-pocket (11%) is higher than HA-brown forest soils-A (8%) and HA-sod-brownzems-A (8.9%); the differences between HA-brown forest-pocket and HA-brown forest soils-A are significant. The higher content of HA-brown forest-pocket indicates a greater number of substituted aromatic fragments or carbonyl-substituted aromatic structures. 

167–185 ppm. This zone reflects carbonyl C of carboxyl, ester, and amide groups characteristic of acidic fragments of organic matter. HA-brown forest soils-A and HA-sod-brownzems-A have similar values (17–17.7%), slightly higher than HA-brown forest soils-pocket (16%). 

185–220 ppm. This range is associated with ketone and quinone carbons. The contribution is low in all samples.

**Figure 1:**
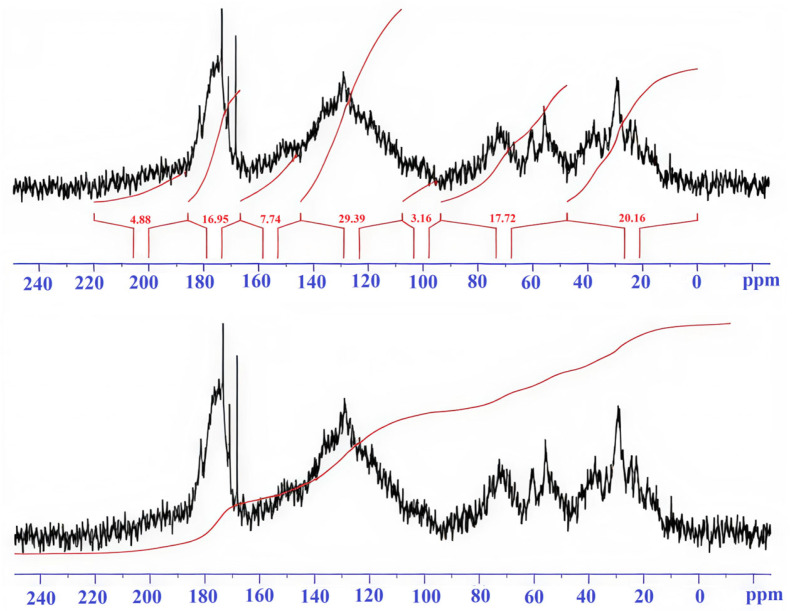
13C-NMR spectrum of the humic acid brown forest soils.

HAs from horizon A of brown forest soils are characterized by a relatively balanced ratio of aliphatic and aromatic structures, reflecting an intermediate degree of humification and moderate aromatization of organic matter. A significant content of aliphatic carbons is observed in the 0–48 ppm (19%) range (Table 2, Figure 1). The 48–90 ppm (17.7%) range corresponds to aliphatic carbon atoms bonded to oxygen or nitrogen, indicating the presence of alcohol, ester, and amine functional groups. In the 90–108 ppm (≈3%) range, the proportion of acetal carbons is minimal, indicating an insignificant content of carbohydrate fragments. The main contribution of the spectrum (29.5%) is observed in the range of 108–145 ppm, where aromatic structures predominate, forming the stable framework of the humic substance. Signals in the region of 145–167 ppm (8%) reflect the presence of substituted aromatic carbons (phenolic and carbonyl), which impart reactivity to the substance. The high percentage of carboxyl, ester, and amide carbon atoms (17.4% at 167–185 ppm) indicates significant oxidation and acidity of the HAs. In the latter range of 185–220 ppm (6%), ketone and quinone structures typical of mature forms of humic substance appear. Thus, the HAs of horizon A of brown forest soils possess a developed system of oxygen-containing groups, a combination of aliphatic and aromatic components, and a moderate degree of condensation, indicating a transitional stage of humus between fresh organic matter and highly aromatic forms.

**Figure 2:**
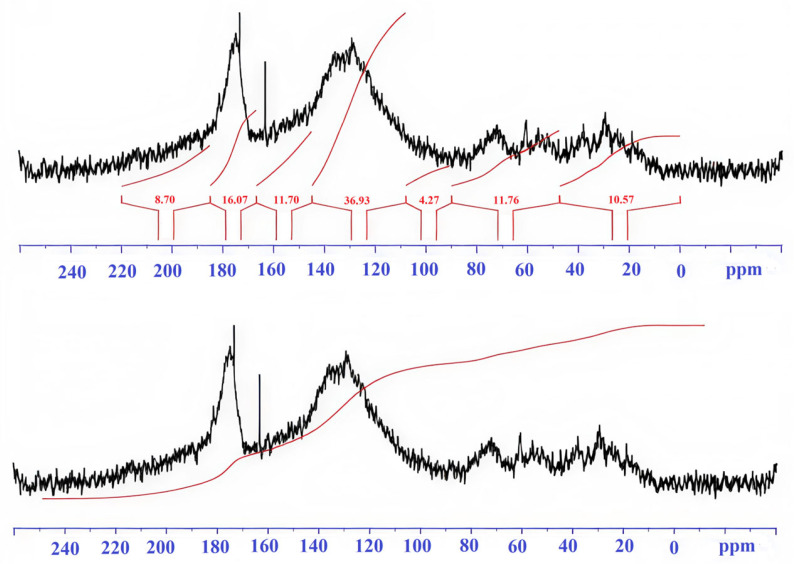
^13^C-NMR spectrum of the humic acid in brown forest soils pocket.

HAs from the brown forest soils pocket, according to their ^13^C NMR spectra, are characterized by a predominance of aromatic structures and a high content of oxygen-containing functional groups. The proportion of aliphatic carbons (0–48 ppm) is only about 11.6%, indicating a limited number of methylene chains and low hydrophobicity (Table 2, Figure 2). The low contribution of acetal carbon atoms (4%) confirms the weak development of polysaccharide residues. The main part of the signal (37.7%) is due to aromatic structures (108–145 ppm), indicating a high degree of condensation and stability of the organic matter. In the 145–167 ppm range, approximately 11% of substituted aromatic carbons containing phenolic and carbonyl groups were recorded. In the 167–185 ppm range (16%), a significant amount of carboxyl, ester, and amide carbons was noted, providing their high reactivity. The presence of ketone and quinone structures (8%) in the 185–220 ppm range indicates an advanced stage of oxidation and the maturity of the humic substance. The HA-brown forest soils-pocket soils differ in their spectra: a higher proportion of carbon in the region of average chemical shifts and reduced low-frequency components—this may reflect differences in the origin of OM and the degree of humification.

**Figure 3:**
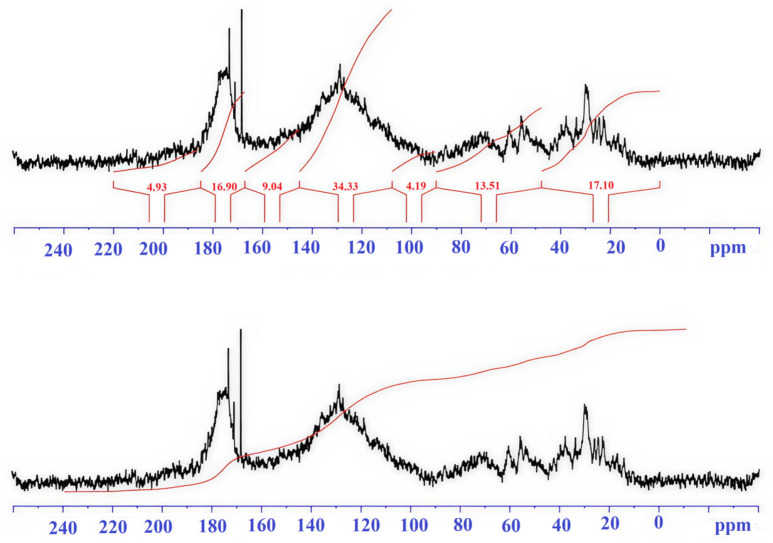
^13^C-NMR spectrum of HAs of sod-brownzems-A.

The humic acid spectrum of sod-brownzems soil (Table 2, Figure 3) is characterized by a significant share of the aromatic component, indicating a high content of aromatic rings and phenolic groups–structures characteristic of HAs. Aliphatic carbon accounts for approximately 17%, indicating the presence of chains and side groups. A significant portion of the carbon is associated with C-O and C-N functions (12.5% and 3.5%), indicating the presence of alcohols, amines, and acetals—common functional groups. The content of carboxyl, ester, and amide groups (17.7%) confirms the acidic nature of humic acid and the possibility of bonding with nitrogen. HAs of these soils is characterized by a relatively small amount of ketones and quinones (6.0%).

**Figure 4:**
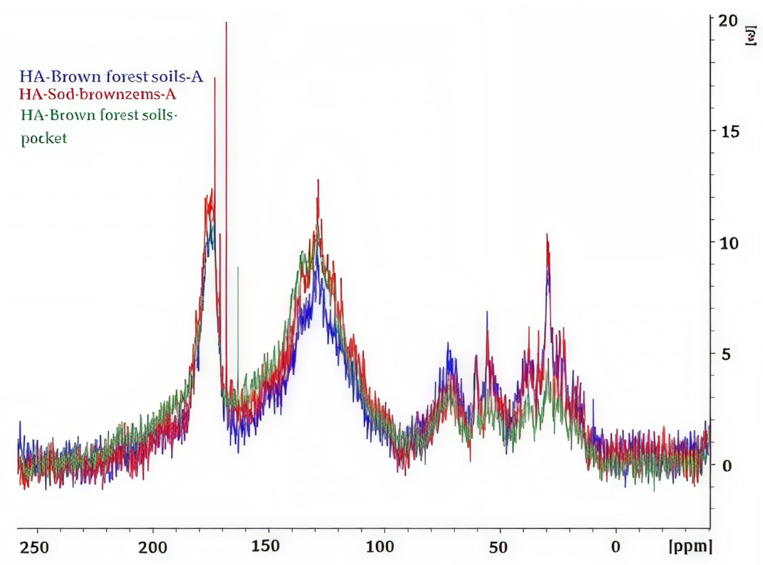
Comparison of ^13^C-NMR spectra of the studied HAs.

Figure 4 shows the ^13^C-NMR spectra of the three studied samples for visual comparison. It is evident that the relative signal intensity is reduced: at the aliphatic carbon atoms of the HA-brown forest soils-pocket (green curve) and at the aromatic carbon atoms of the HA-brown forest soils-A.

Thus, the main differences between the HAs are manifested in the aromatic range (108–167 ppm): HA-brown forest soils-pocket is characterized by maximum aromaticity and substituted aromatic structures, while HA-brown forest soils-A and HA-sod-brownzems-A have a greater contribution of aliphatic and oxygenated units, indicating a less condensed and more functionalized organic matrix.

### 2.4. Elemental Composition

**Figure 5:**
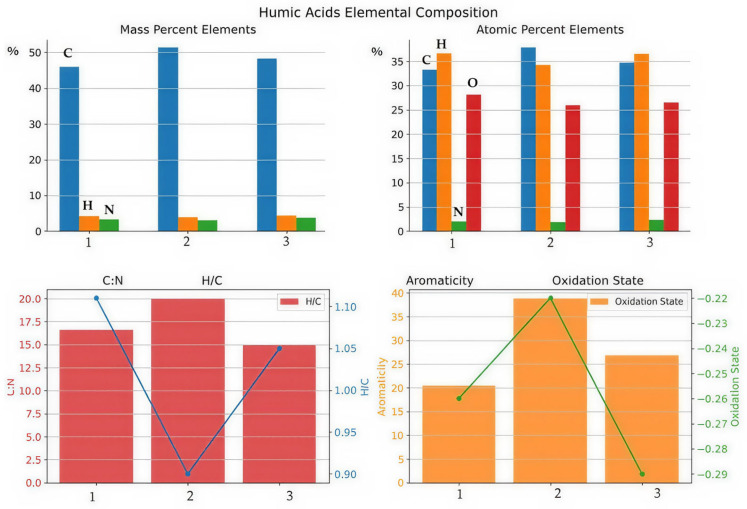
Elemental composition of HAs. Legend: 1—HA-brown forest soils, 2—HA-brown forest-pocket, 3—HA-sod-brownzems.

Significant differences in the mass fractions of the main elements were revealed in the humic acid composition of the studied soils. The maximum carbon content was observed in HA-brown forest-pocket (51.44%), which indicates a high degree of condensation and development of the aromatic matrix (Figure 5). HA-sod-brownzems-A is characterized by an intermediate value (48.21%), while the lowest carbon content was observed in HA-brown forest soils-A (45.93%). The hydrogen content shows the opposite trend: the highest value was found in HA-brown forest soils-A and HA-sod-brownzems-A (4.26–4.27%), which reflects a more pronounced aliphatic component of the structure. The minimum hydrogen content was found in HA-brown forest-pocket (3.91%), which is consistent with its high aromatization. The highest nitrogen content is found in HA-sod-brownzems-A (3.75%), while the lowest is found in HA-brown forest-pocket (3.00%).

The atomic ratios of the elements also reveal structural differences between the HAs. The highest atomic carbon content is found in HA-brown forest-pocket (37.90%), confirming the predominance of condensed carbon structures; lower values are found in HA-sod-brownzems-A (34.68%) and HA-brown forest soils-A (33.22%) (Figure 5). The atomic fraction of hydrogen varies from 34.26% in the most aromatized HA-brown forest-pocket to 36.64% in HA-brown forest soils-A. The atomic fraction of nitrogen varies from 1.89% (HA-brown forest-pocket) to 2.31% (HA-sod-brownzems-A), reflecting differences in the degree of preservation of nitrogen-containing fragments.

The C/N ratio is an indicator of the depth of organic matter transformation. A value of 20.0 for HA-brown forest-pocket is the highest and indicates the deepest humification, loss of nitrogen components, and the dominance of stable carbon structures. Lower values were found in HA-brown forest soils-A (16.6) and HA-sod-brownzems-A (15.0), indicating a higher proportion of young nitrogen-containing structures in the latter two samples.

The H/C ratio ranges from 0.90 in HA-brown forest-pocket to 1.11 in HA-brown forest soils-A, with an intermediate value (1.05) observed in HA-sod-brownzems-A. The lowest H/C ratio in HA-brown forest-pocket indicates the highest aromaticity, while aliphatic components predominate in HA-brown forest soils-A. A ratio of 1.05 for HA-sod-brownzems-A is closer to aliphatic structures and reflects a lower degree of aromatization. The highest value of 1.11 in HA-brown forest soils-A indicates a significant proportion of aliphatic fragments and low condensation.

The degree of benzenoidity (Figure 5) of HAs reflects the extent to which the average organic matter structure possesses an aromatic, “benzenoid” character. High values indicate a significant proportion of condensed aromatic rings, while low values indicate a predominance of aliphatic and less aromatic fragments. The degree of benzenoidity clearly reflects differences in the degree of aromatization of HAs. The minimum value for HA-brown forest soil-A is 20.5, the average for sod-brownzems HAs is 26.8, and the maximum for HA-brown forest-pocket is 38.8, indicating a progressive increase in aromatic condensation. As benzenoidity increases, the H:C ratio naturally decreases, consistent with Van Krevelen’s rule: increased aromaticity is accompanied by hydrogen loss and a decrease in the degree of saturation of the carbon skeleton. This confirms that the ternary series of samples reflects a sequential transition from less condensed to more condensed aromatic structures. The oxidation state of HAs indicates the degree to which the carbon skeleton of the molecule is, on average, “oxidized” or “reduced.” Negative values indicate a reduced structure, i.e., a high contribution of saturated fragments and a relative deficiency of oxygen-containing functional groups. All studied HAs are characterized by negative oxidation states, indicating the predominance of reduced forms of carbon. Sod-brownzems’ humic acid is the most reduced (−0.29), which corresponds to the limited oxidation conditions of taiga soils. HA-brown forest soil-A has a value of −0.26, occupying an intermediate position. HA-brown forest-pocket is the least reduced (−0.22), which may reflect a higher proportion of oxygen-containing groups or more advanced processing of organic matter. The closer the oxidation state is to zero, the higher the relative oxidation of organic matter. A value of −0.22 indicates that this humic acid is the least reduced and most oxidized of the samples. This is consistent with its high benzenoid content: more condensed and aromatic structures are often accompanied by more pronounced oxygen functionalization.

The studied HAs differ in the mass and atomic composition of their elements, as well as in their atomic ratios, benzenoid content, and oxidation state. These differences reflect variations in soil formation conditions, humification intensity, and the depth of structural processing of organic matter.

The combined results of ^13^C-NMR spectroscopy and elemental analysis of HAs showed that HA-brown forest-pocket has the most mature, condensed, and aromatized structure, while HA-brown forest soils-A is characterized by the lowest aromatization. HA-sod-brownzems-A occupies an intermediate position, combining high aromaticity with a comparatively elevated nitrogen content and somewhat less pronounced structural condensation.

## 3. Discussion

Brown forest soils are characterized by the formation of forests with medium stand density and moderate species diversity. Dahurian larch, aspen, and broadleaf birch predominate here, indicating adequate moisture and nutrient supply. The understory, including Dahurian rhododendron, cotoneaster, and rosehip, reflects stable but not dense vegetation development. The grass–dwarf shrub layer, dominated by lingonberry, and a sparse moss–lichen cover indicate stable but relatively moderate moisture and heat conditions. Overall, the vegetation of brown forest soils forms a balanced forest ecosystem with sustainable functioning and moderate productivity. The Corg content of brown soils in the upper horizon of the soil layer corresponds to the values indicated in the National Atlas [22] and the data of Kulikov et al. [23]. A sharp decrease in Corg in the 20–30 cm layer to 0.77% and the presence of elevated values in humus pockets (up to 5.1%) indicate cryogenic redistribution of carbon and its immobilization in local reservoirs, which is not noted in literary descriptions, where the profile is more stable.

In the studied soils, the Corg content is within the reference values for this soil type compared to other regions. For example, in the brown forest soils of the Western Caucasus it is 4.3 [24], in the brown forest soils of the Republic of Armenia Corg reaches 6.5% [25], in the north of Iran the Corg content in soils is 3.0–5.5% [26], in the brown forest soils of the Kuznetsk Alatau it is from 3.5 to 5.2% [27], and in the dark-colored brown forest soils of Karelia it is 2.3–3.5% [28]. The soil pH is slightly acidic, consistent with the characteristics of slightly unsaturated brown soils (up to 90% base saturation) from the National Atlas. The high content of exchangeable Ca and Mg (35.9–36.6 cmol (eq)/kg) confirms adequate nutrient availability and is consistent with the concept of high absorption capacity (≈12–40 mol (eq)/100 g).

The coefficient of variation in Corg content (15–30% in the profile and 29–50% in pockets) reveals pronounced spatial heterogeneity, which is associated with cryogenic cracks. In the literature [29], such heterogeneity is primarily associated with changes in stoniness, whereas in our case, it is due to cryogenic influence.

Thus, brown forest soils have satisfactory physical-chemical properties. 

Sod-brownzems (Leptic Cambisols Skeletic) form beneath larch forests. The dominant species is Dahurian larch, which ensures the stability of this type of ecosystem. The vegetation cover is sparse, with the herbaceous layer primarily consisting of moisture-loving and cold-hardy species (reed grass, sedge, burnet, fireweed, etc.). The presence of shrubs and a moss–lichen layer indicates moderate but stable vegetation development. Low and sparse stands with thin trunks indicate unfavorable conditions for productive forest growth. Overall, these soils support stable but low-productivity forest communities adapted to the cold climate of Transbaikalia.

In the upper horizon (0–10 cm), they are characterized by a high content of Corg—6.03% and nitrogen—0.59%, which is consistent with data from the National Atlas of Russia [22], which indicates a humus content of 4–8% with a sharp decline down the profile. High organic matter levels reflect the slow mineralization of plant residues in cold and wet conditions that promote humus accumulation. The pH of the environment is slightly acidic (pH 5.70) in sod-brownzems; however, in deeper horizons (up to pH 5.10), acidity increases due to leaching of bases and weakened humus formation.

As the carbon and nitrogen content decreases (to 0.50% and 0.20% at a depth of 20–30 cm), a transition to the mineral part of the profile appears, accompanied by a drop in the exchangeable base content from 31.50 to 6.90 cmol (eq)/kg. This indicates a decrease in the saturation with Ca^2+^ and Mg^2+^ cations and a reduction in the soil’s buffering properties. According to the National Atlas of Russia [22], base saturation in sod-brownzems saturated soil types reaches 80–100%- therefore, the studied samples belong to moderately saturated varieties, characteristic of more acidic boreal soils.

The profile is characterized by a gradual decrease in the physical clay content and an increase in coarse sand to 60–80% in the lower horizons. This structure promotes good drainage and aeration. As noted by Kulikov et al. [23], up to 67–69% of the pore space in horizons BC and C is occupied by large air-bearing pores, which ensure the effective outflow of excess moisture and stabilization of the humidity-thermal regime.

In general, sod-brownzems exhibit characteristic zonality: high organic matter content in the humus horizon, a sharp vertical gradient of carbon and nitrogen, increasing acidity down the profile, and a gradual decrease in absorption capacity. These properties, along with favorable aerobic conditions in the lower part of the profile, reflect the specific nature of the formation of cold boreal forest soils on coarse-grained eluvium-coluvium and confirm the dependence of their chemical and physicostructural characteristics on the composition of parent rocks (National Atlas of Russia) [22,23].

Thus, sod-brownzems are characterized by satisfactory physical–chemical properties.

Analysis of HAs by ^13^C-NMR spectroscopy allowed us to identify chemical shifts associated with the carbon atoms of various functional groups and molecular fragments of HAs [30].

HA-brown forest soils-A (Figure 5) is characterized by reduced intensity in the aromatic region, which may be due to a reduced proportion of aromatic carbons and less pronounced aromatic structures, such as lignin-like or condensed aromatic fragments. The weakening of the signals can also be explained by increased interaction of aromatic compounds with the mineral phase, which causes broadening of the spectral lines and a decrease in their amplitude, as well as differences in the degree of condensation and polymerization of aromatic systems. Furthermore, this manifestation may reflect the characteristics of the organic matter sources and differences in their transformation pathways.

The HA-brown forest soils-pocket (Figure 4) exhibits reduced intensity in the aliphatic region, which may be due to a lower number of alkyl fragments in the organic matter compared to other samples. This manifestation may also indicate a higher degree of degradation of easily mobile organic matter or a lower content of fresh lipid and cellulosic components.

HAs from the sod-brownzems (Figure 4) have a high content of aromatic compounds, indicating a high content of lignin and strong condensation of aromatic fragments. Aliphatic carbon (approximately 17%) is represented by chain and side groups, ensuring the mobility of the structure. A significant portion of the C-O and C-N spectral range is associated with alcohols, amines, and acetals, facilitating interactions with minerals. The high content of carboxyl, ester, and amide groups (17.7%) provides acidity and interactions with nitrogen-containing compounds. Ketones and quinones (6.0%) reflect the stability of the substance.

The degree of organic matter transformation was clarified based on a quantitative analysis of signal ranges in the ^13^C-NMR spectra. According to generally accepted criteria based on the works of Baldock and Preston [8], Kögel-Knabner [1], and Simpson et al. [10], an increase in the proportion of aromatic structures (160–110 ppm) and a simultaneous decrease in the contribution of O-alkyl carbon (110–45 ppm) is interpreted as an indicator of a higher degree of humification. The 0–48 and 48–90 ppm ranges reflect the content of alkyl and O-alkyl fragments sensitive to destruction during humification. The 108–167 ppm ranges correspond to aromatic structures, the increase in whose proportion is associated with an increase in the degree of condensation and aromatization. The 167–220 ppm ranges characterize carbonyl and carboxyl groups, reflecting the degree of humus oxidation. The ratios between these ranges (e.g., alkyl/aromatic signals, O-alkyl/alkyl) are used in the literature to assess the stage of humification and structural changes. Using these threshold ratios allows for an objective characterization of the degree of structural ordering and the depth of organic matter transformation in the studied samples, ensuring comparability of the obtained data with the results of other studies.

HA-brown forest soils are characterized by moderate aromaticity (37.48%), indicating a moderate degree of humification. The elevated proportion of O-alkyl-C (17.69%) indicates a significant presence of carbohydrates and partially decomposed plant residues. Aliphatic carbon (18.90%) and a relatively high carbonyl carbon (22.96%) reflect a combination of lipid fragments and oxidized structures.

HA-brown forest-pocket soils have the highest aromaticity (48.65%), indicating an advanced stage of humification and the accumulation of condensed aromatic structures. The minimal O-alkyl-C content (11.21%) indicates almost complete carbohydrate breakdown, and the low proportion of aliphatic carbon (11.56%) reflects a decrease in the amount of easily degradable compounds.

HA-sod-brownzems are characterized by an aromaticity of 43.20%, which exceeds the values of typical brown forest soils but is lower than pockets, indicating an intermediate degree of decomposition. Moderate amounts of O-alkyl-C (12.46%) and aliphatic carbon (17.24%) reflect the preservation of some primary biomolecules. A consistently high proportion of carbonyl structures (23.71%) is typical of humic substances formed under conditions of intense oxidation. Thus, the structure of HAs shows a balance between humification and the preservation of primary organic fragments, characteristic of sod-brownzems conditions.

The HAs of pockets in brown forest soils are the most humified and structurally condensed material. The lowest degree of humification is characteristic of HAs in brown forest soils, where a significant proportion of fresh plant residues is preserved. Sod-brownzems occupy an intermediate position, combining the features of both types.

The literature contains studies examining the characteristics of HAs in Arctic soils. For example, a Cryosol study in the Yamalo-Nenets Autonomous Okrug noted that HAs are characterized by a predominance of aliphatic structures. The content of C,H-substituted fragments (0–47 ppm) reaches 49.1%, and their proportion increases down the profile. O,N-substituted aliphatic structures account for up to 20%, but are unevenly distributed. Aromatic components are also present in significant quantities and increase with depth to 20.7% [21].

As noted in a number of studies, the high aliphatic content of HAs in organogenic horizons is associated with the accumulation of labile carbon from poorly decomposed plant residues [31]. The predominance of aliphatic structures is explained by the deficiency of lignin in tundra vegetation (mosses and lichens), which serves as the main source of organic matter [32].

Moreover, according to literature data, the increase in the proportion of aromatic (108–164 ppm) and carboxyl (164–183 ppm) fragments in HAs reflects lignin humification processes and the formation of structures more resistant to biodegradation, characteristic of mineral horizons [33]. The overall low aromaticity of humic substances in Arctic soils is associated with increased humidity, anaerobic conditions, and low microbial activity, which slows down humification processes [34].

According to literature data, organogenic soil horizons are rich in carbohydrate, amino, and methoxyl groups, the proportion of which decreases with depth due to destruction and oxidation [35]. HAs are characterized by the dominance of aliphatic fragments, while in the soils of the southern zones there is an increased content of aromatic structures due to differences in humification precursors and more favorable bioclimatic conditions [30,35].

HAs play a key role in the long-term stabilization of Corg in soils, as they form strong, difficult-to-decompose complexes with the mineral component and provide chemical protection of organic matter from microbial degradation. Thus, HA-brown forest soils-A plays an intermediate role in the carbon stabilization process. Its HAs are in the transitional stage of humification and contain both aliphatic and aromatic structures, which ensures partial stability of organic matter while maintaining its capacity for biotransformation. The HA-brown forest-pocket is a powerful carbon stabilizer: its high aromaticity and developed system of oxidized and polycondensed structures make carbon difficult for microbial degradation. As a result, most of the Corg is fixed in stable forms. HA-sod-brownzems-A occupies an intermediate position between HA-brown forest soils-A and HA-brown forest-pocket, but is closer to the latter in terms of aromatization. High oxidation and a developed system of functional groups contribute to the formation of stable organomineral complexes, which enhance carbon retention and long-term stabilization.

Thus, the carbon stabilization stress increases in the series HA-brown forest soils-A → HA-sod-brownzems-A → HA-brown forest-pocket, reflecting the transition from less stable, bioavailable forms to deeply condensed and long-lasting carbon compounds.

Elemental analysis revealed that the carbon content was highest in the HA-brown forest-pocket sample (51.44% by weight), indicating a higher level of organic matter and a potentially more stable carbon skeleton. The HA-sod-brownzems-A sample had slightly lower carbon content (48.21%), but a more balanced carbon-nitrogen ratio was observed.

The C:N ratio varied from 15 in the sod-brownzems sample to 20 in the brown forest-pocket sample, reflecting differences in nitrogen bioavailability. A low C:N ratio indicates better organic matter quality for microbial decomposition.

The H:C ratio reflects the degree of saturation of organic compounds: values around 1 indicate a moderate content of saturated hydrocarbon chains. This ratio is lower in the HA-brown forest-pocket sample (0.90), which may indicate a more pronounced aromatic character of the substance.

A cross-analysis of the degree of benzenoidity and oxidation state shows that changes in the aromaticity and oxidation state of HAs reflect a directional increase in their structural complexity. An increase in SB from HA-brown forest soil-A to the HA-sod-brownzems and HA-brown forest-pocket indicates an increase in the proportion of condensed aromatic fragments, which is accompanied by a decrease in H:C and confirms increased aromatization. We want to note that the HA-brown forest-pocket has a higher degree of aromaticity, so with a slightly lower O and N content and a reduced H content, its calculated oxidation state is higher. This is due to the low H/C ratio and more condensed structure, rather than an actual increase in oxidation. In other words, the increase is due to the calculated characteristics and structure, not due to a higher content of oxygen functional groups.

Modern studies of Cambisols indicate that their organic matter is characterized by relatively high carbon and nitrogen contents, associated with intensive accumulation of forest litter and active humus formation [36]. Elemental analysis studies indicate that brown forest soil are characterized by relatively low hydrogen concentrations and an increased proportion of oxygen in the humic substances, which is associated with the accumulation of carboxyl and phenolic groups with increasing depth in the profile [37].

The obtained data on the elemental composition are completely consistent with the results of ^13^C-NMR spectroscopy, mutually confirming the identified structural features and the degree of transformation of SOM. In general, the differences between the three types of HAs reflect the influence of soil-forming factors. The higher the aromatization and condensation, the greater the stability of the humic structures; conversely, increased oxidation and aliphaticity indicate less mature humus and an active biodegradation cycle. Thus, the properties of HAs serve as an indicator of the direction of organomineral processes in each soil type and allow one to assess the stability of organic matter in the corresponding ecosystems. Thus, the identified differences reflect the gradation of HAs from less stabilized and more oxidized (HA-brown forest soils-A) to more aromatized and structurally stable (HA-brown forest-pocket), while the HAs of the HA-sod-brownzems-A occupy an intermediate position with pronounced nitrogen saturation.

Low-temperature conditions determine the structural properties of HAs. Slow microbial degradation of lignin and phenolic compounds preserves aromatic nuclei and limits their oxidative degradation. This promotes increased aromatization and the accumulation of condensed, stable aromatic fragments. At the same time, incomplete decomposition of plant residues increases the contribution of oxygen-containing groups, including carboxyl, carbonyl, and phenolic structures. Under such conditions, nitrogen-containing fragments also accumulate, reflecting the low degree of conversion of protein and amine compounds. Overall, low temperatures produce HAs with a youthful structure, a high proportion of aromatic components, and an increased content of oxygenated functional groups, ensuring their long-term stability in cold ecosystems.

Studying the humic acid structure of brown forest soils and sod-brownzems of the southern Vitim Plateau is of significant ecological importance. The spectral characteristics of HAs reflect the degree of Corg stabilization and allow us to assess its retention in a sharply continental climate. These data clarify the role of soils in the regional carbon cycle and help predict the sensitivity of ecosystems to changes in temperature and humidity. The qualitative characteristics of humus determine the stability of soil fertility, the ability of soils to retain nutrients and moisture, and their resistance to degradation. Using ^13^C-NMR parameters as indicators of soil processes enhances the ability to monitor anthropogenic impacts and assess the consequences of climate fluctuations. Thus, the results of this study expand our understanding of the mechanisms of organic matter storage and transformation and clarify the ecological role of soils in cold regions in maintaining the carbon balance and ecosystem functioning.

This study is unique in that it provides the first ^13^C-NMR data on HAs in two types of forest soils and in humus pocket in the cold zone of the Vitim Plateau, allowing us to clarify their aromaticity and stability SOM. These data reveal the duration of carbon retention in boreal soils and contribute to our understanding of the role of cold soils in the global carbon cycle, including their resistance to mineralization and their possible response to climate warming

## 4. Materials and Methods

**Figure 6:**
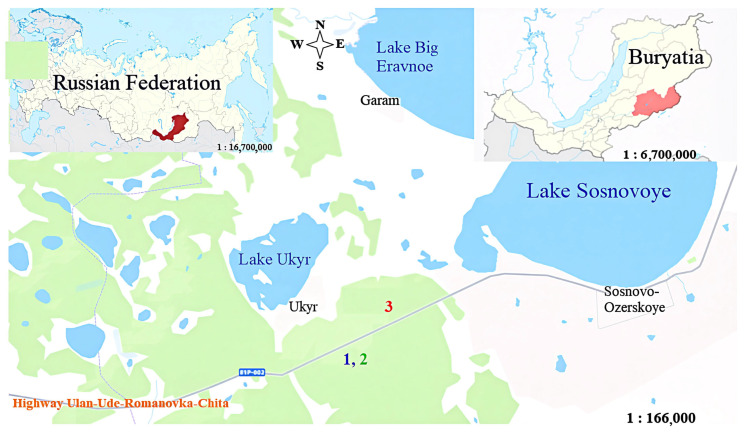
Map-scheme of the location of experimental sites. Legend: 1—brown forest soil; 2—brown forest soil with a humus pocket; 3—sod-brownzems.

The objects of this study were brown forest soils and sod-brownzems of the southern Vitim Plateau (Figure 6). To ensure clarity and unambiguity in the designation of soil samples, the following designations for soil humus preparations are used in the text below: “HA-brown forest soils-A”—a soil sample taken from the humus-accumulative horizon of the brown forest soils. “HA-brown forest-pocket”—soil material collected from the humus pocket of the brown forest soils. “HA-sod-brownzems-A”—a soil sample from the humus-accumulative horizon of the sod-brownzems.

The soils were classified according to Egorov and co-authors [38] and IUSS Working Group WRB [39].

### 4.1. Soil Sampling

Fieldwork was conducted in the Darkhituy area during the peak of active layer thaw and vegetation growth. Within the experimental sites, relief areas were selected that most fully reflected the diversity of soils. Three soil profiles were laid for each soil type, for a total of six profiles: three brown forest soils profiles with pockets and three sod-brownzems profiles. All soil samples were collected in triplicate across genetic horizons. Samples were taken only from undisturbed areas to ensure their representativeness. The main physicochemical soil parameters were determined in triplicate (n = 3).

To isolate humic acid compounds and further analyze the structural composition of HAs, samples were collected from the upper humus-accumulative horizons and soil material from the humus pocket. To isolate humic compounds, an average sample was formed for n = 3. Equal-weight average samples were collected from each profile from the upper horizon (n = 3) (0–20 cm). The sampled material was freed of roots, large inclusions, and plant debris. All samples were then combined into a single container and thoroughly mixed until a homogeneous mass was obtained, followed by the formation of a medium sample. The soil was crushed and sieved through a 2 mm sieve to obtain a fine-grained fraction, ensuring standardized soil preparation and comparability of analytical data according to international standards.

Immediately after collection, visible large plant debris, including root fragments, stems, twigs, moss, and large pieces of bark, were manually removed from the sample. This preliminary cleaning is necessary to prevent the introduction of fresh organic matter, which is not considered humus and can distort the spectral characteristics of HAs. Before sifting, the soil was thoroughly crushed by hand to break up large clumps without altering the natural structure of the fine aggregates.

Sifting was carried out in an air-dry state, which prevents soil compaction and ensures consistent particle passage through the sieve. The sieve was rinsed and cleaned before each use to prevent cross-contamination between samples. After sifting, the remaining particles were carefully inspected to remove any stray organic matter that might have remained after the preliminary cleaning. The sample that passed through the sieve was visually inspected and, if any were detected, they were further removed with tweezers.

During the study, the influence of vegetation type and topography was taken into account during the planning and stratification of field plots. Each sampling point was assigned to a specific phytotype, recorded in a field log, and considered a key source of variation in humus composition. The location of the points within the microrelief—whether on a peak, slope, or depression—was also recorded, as topography influences moisture redistribution and the rate of decomposition of organic matter. This stratification ensured representative sampling and allowed for the accurate interpretation of differences in the spectral characteristics of HAs without confounding the effects of vegetation, elevation, and topography.

### 4.2. Analyses

Total C and N contents and elemental composition of HAs were determined on a Perkin Elmer CHNS/O Series II analyzer (Norwalk, CT, USA) at the Institute of General and Experimental Biology, Siberian Branch, Russian Academy of Sciences (Ulan-Ude). Due to the absence of carbonates in the studied soils, the total carbon value determined using an elemental analyzer was reasonably interpreted as the content of Corg, which corresponds to generally accepted practice for non-carbonate soils [40]. pH values were measured potentiometrically in aqueous suspensions, soil:water ratio (1:2.5). Particle size distribution was determined by sedimentation, via the pipette method [41]. Carbonates were not determined because there was no reaction to boiling from 10% HCl. All analyses were performed in triplicate; the results are shown in Table 1.

HAs were extracted from soil samples according to the standardized procedure recommended by the International Humic Substances Society (IHSS), taking into account modifications introduced by R.S. Vasilevich et al. [42].

Nuclear magnetic resonance studies were performed using a Bruker (Billerica, MA, USA) AV-600 NMR spectrometer (resonance frequencies of 600.18 MHz for 1H and 150.93 MHz for ^13^C) at the Novosibirsk Institute of Organic Chemistry, Siberian Branch of the Russian Academy of Sciences (Novosibirsk, Russia). Literature data were used to select the parameters for recording ^13^C-NMR spectra and interpret the results [43]. Samples for recording ^13^C-NMR spectra were prepared by dissolving sample in a NaOH solution in deuterated water (D2O). The insoluble residue was separated by centrifugation after dissolution; the resulting precipitate was not used in further measurements. The solutions were not concentrated, as drying the samples could alter the structure of the humic substances. All spectra were recorded directly from the solutions obtained after separation of the insoluble residue. Standard 5 mm Bruker glass ampoules designed for high-field spectrometers were used for NMR measurements. The solutions were carefully transferred into these ampoules. This procedure ensures reproducibility of sample preparation and NMR conditions.

^13^C-NMR spectra were recorded with suppression of spin-spin interaction with protons (proton decoupling). For decoupling, the inverse discontinuous decoupling method was used, with the decoupling generator turned on only during signal reading and turned off for the relaxation delay period. The spectrum sweep width was approximately 45,450 Hz, the free induction decay signal (FID) recording time was 0.72 s, the interval between pulses was 4 s, the pulse width was 45°, and the spectrum accumulation duration was 15–22 h. The Fourier transform was performed with preliminary exponential weighting of the FID signal with a time constant equivalent to a line broadening of 20 Hz. Spectrum phasing was performed manually. Baseline alignment was performed using the extreme signal-free portions of the spectrum (<0 and >220 ppm) using a linear function. The integration of the intensity of groups of signals located in separate spectral regions was performed, the division into which was carried out in accordance with literary analogies of the characteristic spectral regions of various structural fragments [43].

Data interpretation was performed by assigning ^13^C-NMR signals to functional groups and evaluating their relative integrated intensities, which reflect the proportion of carbon atoms in the corresponding structural fragments of HAs. The following procedures were used: assigning chemical shift ranges to carbon classes (aliphatic, oxygen-containing, aromatic, etc.), integrating signals in specified spectral regions to obtain relative areas, normalizing the integrals between samples under identical recording conditions, and accounting for possible intensity distortions associated with differences in T1, line width, and signal overlap.

The resulting relative integrals were used for a comparative analysis of the humic acid composition and to discuss differences in the proportions of aliphatic and aromatic fragments between samples.

To improve the reliability of the integration results, multiple independent phasing and baseline alignments of the spectra were performed. The relative integral signal intensity obtained as a result of each independent spectral processing and the final result obtained after statistical processing are presented in Table 2.

A 19 mg sample of HA-brown forest soils-A was dissolved in 0.6 mL of 0.1 M NaOH in D2O by analogy with the literature [43]. The ^13^C-NMR spectrum was accumulated for 22 h (16,128 scans). The obtained spectrum (Figure 1) was assessed as insufficiently accumulated for interpretation. In this regard, the concentration of substances in solutions for accumulation of ^13^C-NMR spectra was increased. A 55.3 mg sample of HA-brown forest soils-A was treated with 0.6 mL of 0.51 M NaOH in D2O. The substance dissolved gradually, forming a deep dark brown solution. The dissolution process was monitored visually by the presence of solid particles on the bottom and walls of the vessel after draining the solution. The presence of solid particles did not affect the recording of spectra, since they did not enter the NMR tube. The ^13^C-NMR spectrum (Figure 2) was collected over 22 h (16,898 scans). The acceptable stability window for ^13^C-NMR accumulation is 24 h.

A 98 mg sample of HA-brown forest-pocket was dissolved in 0.9 mL of 0.51 M NaOH in D2O. The substance gradually dissolved, forming a deep dark brown solution. 0.6 mL of the resulting solution was placed in an NMR ampoule. The ^13^C-NMR spectrum of the resulting solution (Figure 3) was collected over 15 h (11,200 scans).

A 58 mg sample of HA-sod-brownzems-A was dissolved in 0.6 mL of 0.51 M NaOH in D2O. The substance gradually dissolved, forming a deep dark brown solution. The ^13^C-NMR spectrum of the resulting solution (Figure 5) was collected over 23 h (17,664 scans).

### 4.3. Calculations and Statistical Analysis

Microsoft Excel and Statistica 12 programs were used for statistical processing of the data.

### 4.4. Conditions for the Formation of Soils and Plants

According to geocryological zoning, the Yeravninskaya Basin is located in the transition zone between continuous permafrost and sporadic permafrost. Permafrost thickness in this region ranges from 70 to 100 m, with its upper boundary occurring at a depth of approximately 1.5–3.0 m below the surface. According to some data, the permafrost level is recorded at a depth of approximately 270 cm, confirming the transitional nature of the permafrost between continuous and sporadic permafrost. The Vitim Plateau is characterized by the widespread distribution of loose Quaternary sediments—eluvium, colluvium, and alluvium—which, due to active physical weathering, form a continuous blanket covering watersheds, mountain slopes, and intermontane depressions.

Brown forest soils are common on the plains of the southern Far East and in the mountains of the Caucasus, Altai, Transbaikalia, and Sikhote-Alin. They form in a moderately warm, humid climate under broadleaf and coniferous-broadleaf forests on heavy loamy lacustrine-alluvial deposits and loamy-gravelly derivatives of dense silicate pores [22].

The main soil-forming processes are litter formation, humus accumulation, clay formation, coagulation, and biogenic structuring.

The morphological structure of the profile is AO-A-Bm-BmC-C. In the profile of brown forest soils, beneath a thin (1–3 cm), poorly decomposed litter layer (O), a dark gray organomineral horizon (AO) (1–5 cm) is often distinguished. It consists of a mechanical mixture of plant residues of varying degrees of decomposition and mineral grains. Beneath the AO horizon lies a grayish-brown humus horizon (A) 10–30 cm thick, with a granular-cloddy structure and a high concentration of tree and herbaceous roots. Below, a denser clayey structural-metamorphic horizon Bm with a thickness of 20–30 cm stands out, sometimes with weak signs of illuviation of sesquioxides and clayey matter, brown in color, with a nutty-lumpy structure, gradually turning into the parent rock C. The thickness of the profile fluctuates between 30–50 cm on highly rubble deposits; on highly weathered rocks and flattened relief elements in brown soils, signs of podzolization appear.

Sod-brownzems are common in the lowland and mountainous regions of the southern Central Siberian Plateau, Transbaikalia, and the Sayan Mountains. They form in a sharply continental humid and subhumid climate, primarily under southern taiga coniferous forests of mixed vegetation on sandy loam and light loamy, gravelly silicate rocks of varying composition [22].

The main soil-forming processes are litter formation, humus accumulation, metamorphic ferrugination, and biogenic structuring.

The morphological structure of the profile is AO-A1-Bm-BmC-C. In the sod-brownzems profile, beneath loose, poorly decomposed forest litter (AO) 1–3 cm thick, the humus horizon A is brownish-gray in color, has a lumpy-powdery structure, and contains a high concentration of tree and herbaceous roots. It gradually gives way to a structural-metamorphic horizon (Bm) 20–40 cm thick, pale-brown in color, with a lumpy structure, often containing inclusions of underlying rock debris, the lower edges of which may contain fragmentary thin iron films. The amount of lithic material increases in parent rock (C).

The question of the nature of permafrost (permafrost-affected or seasonally frozen) in sod-brownzems must be determined on a case-by-case basis. Apparently, the influence of lithology and profile drainage on the degree of permafrost is so great that within the continuous permafrost zone, there are areas of infiltration taliks that coincide precisely with the area of these soils.

N.A. Nogina [44] identified sod-brownzems as a subtype within the permafrost-taiga soil type as a typomorphic formation of the lower taiga belt, directly bordering basin landscapes. The nomenclature of soils with a poorly differentiated brown profile has undergone and continues to undergo dramatic changes [23].

## 5. Conclusions

This study shows that the identified structural features of HAs reflect the specificity of organic matter in permafrost soils of the southern Vitim Plateau. Brown forest soils (Haplic Cambisols) and sod-brownzems (Leptic Cambisols Skeletic) are characterized by high Corg (4.9%) and nitrogen (0.48%) contents in the upper horizons, but differ in their distribution across the profile. In brown forest soils, the Corg content decreases sharply with depth and the presence of humus pockets with high carbon content (5.10%) and exchangeable calcium and magnesium (36 cmol (eq)/kg). Sod-brownzems have a higher Corg content in the upper horizon (6.03%), acidity increases with depth, and the amount of exchangeable calcium and magnesium decreases. Thus, brown forest soils and sod-brownzems possess satisfactory fertility, ensuring their stability under natural conditions. The results of the analysis of the ^13^C NMR spectra of HAs revealed clear differences in the structural organization and maturity of organic matter in the studied soils.

HAs from brown forest soils-A are characterized by an equilibrium ratio of aliphatic and aromatic structures, a developed system of oxygen-containing functional groups, and a moderate degree of condensation. This structure indicates a transitional stage of humification: the organic matter is already partially stabilized but remains capable of biotransformation.

HAs from pockets of brown forest soils are highly aromatic, contain significant amounts of substituted aromatic, carboxyl, and quinone groups, and have a low proportion of aliphatic components. This indicates an advanced stage of humification, with a predominance of polycondensed and oxidized structures, ensuring high stability of organic matter. HAs from sod-brownzems are characterized by a high content of aromatic structures (over 34%), indicating significant condensation and the lignin nature of the organic matter. The presence of a developed structure of functional groups (C–O, C–N, COOH, C=O) confirms a high degree of oxidation, which facilitates the formation of stable organomineral complexes.

Comparison of the samples showed that HAs from brown forest soils A and HAs from sod-brownzems A are at similar stages of humification, but the latter are characterized by higher aromaticity and a lower proportion of aliphatic fragments, indicating a more advanced stage of organic matter transformation. HAs from brown forest soil pockets exhibit the highest degree of aromatization and structural stability.

Thus, a steady increase in the complexity and aromatization of the humic acid structure is observed from the A horizon of brown forest soils to the HA-sod-brownzems-A and further to the HA-brown forest-pocket horizon. Carbon stabilization intensity also increases in this sequence, reflecting a gradual transition from less stable and easily decomposed forms of carbon to more condensed and durable compounds capable of ensuring long-term fixation of Corg in the soil matrix. The elemental composition showed that differences between the HAs clearly reflect their maturity: the maximum C content of 51.44% and the minimum H content of 3.91% and N of 3.00% in HA-brown forest-pocket confirm its highly condensed and aromatized structure. HA-sod-brownzems-A is characterized by intermediate values of C (48.21%, H 4.26%) and a maximum N content of 3.75%, indicating a combination of high aromaticity with a lower condensation depth. HA-brown forest soils-A, with a minimum C content of 45.93%, a maximum H content of 4.27%, and an oxidation state of 0.43, is distinguished by the lowest aromatization and the predominance of aliphatic and oxygen-containing fragments. These ratios (C/N 20.0–15.0 and H/C 0.90–1.11) show that HAs are formed under different humification regimes, which determine their structural maturity and chemical specificity. The obtained data on the elemental composition are completely consistent with the results of ^13^C-NMR spectroscopy, mutually confirming the identified structural features and the degree of transformation of SOM.

## Figures and Tables

**Table 1:** Chemical properties of soils.

Soils	Depth, cm	Corg, %	Total Nitrogen, %	pH	Exchangeable Ca Mg, cmol (eq)/kg
Brown forest soils	0–10	4.90 ± 0.65	0.48 ± 0.02	6.40 ± 0.01	36.60 ± 0.35
	10–20	0.75 ± 0.06	0.21 ± 0.01	6.60 ± 0.01	17.60 ± 0.03
	20–30	0.77 ± 0.09	0.21 ± 0.01	6.60 ± 0.01	17.60 ± 0.03
Brown forest—pocket	0–10	4.90 ± 0.65	0.48 ± 0.02	6.40 ± 0.01	36.60 ± 0.35
	10–20	5.10 ± 0.76	0.35 ± 0.03	6.40 ± 0.02	35.90 ± 0.51
	20–30	3.70 ± 0.92	0.64 ± 0.02	6.40 ± 0.01	35.90 ± 0.51
Sod-brownzems	0–10	6.03 ± 0.40	0.59 ± 0.05	5.70 ± 0.05	31.50 ± 0.34
	10–20	2.03 ± 0.20	0.25 ± 0.02	5.20 ± 0.05	14.90 ± 0.31
	20–30	0.50 ± 0.05	0.20 ± 0.01	5.10 ± 0.04	6.90 ± 0.45

**Table 2:** Relative intensity (%) of signal groups of different structural fragments of HAs.

Spectral Range	0–48 ppm	48–90 ppm	90–108 ppm	108–145 ppm	145–167 ppm	167–185 ppm.	185–220 ppm
Carbon atom type Sample:	aliphatic	aliphatic C-O, C-N	acetal	aromatic	aromatic C-O	carboxyl, ester, amide	ketone, quinone
HA-brown forest soils -A	19	17.7	3.0	29.5	8.0	17.4	6
HA-brown forest soils– pocket	11.6	11	4	37.7	11.0	16	8
HA-sod- brownzems -A	17.2	12.5	3.5	34.4	8.9	17.7	6.0

## Data Availability

The data supporting the findings of this study are not publicly available but are available from the author upon request.

## References

[1] Kögel-Knabner I. (1997). ^13^C and 15N NMR spectroscopy as tools in SOM studies. Geoderma.

[2] Orlov D.S. (1990). HA of Soils and the General Theory of Humification.

[3] Kechaikina I.O., Ryumin A.G., Chukov S.N. (2011). Postagrogenic transformation of organic matter in soddy-podzolic soils. Eurasian Soil Sci..

[4] Kurganova I., Merino A., Lopes de Gerenyu V., Barros N., Kalinina O., Giani L., Kuzyakov Y. (2019). Mechanisms of carbon sequestration and stabilization by restoration of arable soils after abandonment: A chronosequence study on Phaeozems and Chernozems. Geoderma.

[5] Artemyeva Z.S., Danchenko N.N., Kolyagin Y.G., Varlamov E.B., Zasukhina E.S., Tsomaeva E.V., Kogut B.M. (2023). Chemical Structure of Organic Matter of Agrochernozems in Different Slope Positions. Eurasian Soil Sci..

[6] Polyakov V., Nizamutdinov T., Abakumov E. (2024). Molecular Composition of HA of Different Aged Fallow Lands and Soils of Different Types of Use in Northwest of Russia. Agronomy.

[7] Napoletano P., Maselli V., Buglione M., Arena C., Zarrelli A., Fulgione D., De Marco A. (2025). Wild boar grubbing affects soil carbon quantity and fractions under native, reforested and planted vegetation. Catena.

[8] Baldock J.A., Preston C.M. (2006). Chemistry of Carbon Decomposition Processes in Forests as Revealed by Solid-State Carbon-13 Nuclear Magnetic Resonance. Carbon Forms and Functions in Forest Soils.

[9] Rumpel C., Kögel-Knabner I. (2011). Deep SOM—A key but poorly understood component of terrestrial C cycle. Plant Soil.

[10] Simpson A.J., Simpson M.J., Smith E., Kelleher B.P. (2007). Microbially Derived Inputs to SOM—Are Current Estimates Too Low?. Environ. Sci. Technol..

[11] Piccolo A. (2022). Humic substances. A review of their nature and structure. Soil Sci..

[12] Sutton R., Sposito G. (2005). Molecular structure of humic substances and its implications. Environ. Sci. Technol..

[13] Zavyalova N.E., Vasbieva M.T., Fomin D.S. (2022). Elemental composition and structure of humic acids of sod-podzolic soil of a long-term stationary experiment and its virgin analogues. Agrochemistry.

[14] Clemmensen K.E., Bahr A., Ovaskainen O., Dahlberg A., Ekblad A., Wallander H., Stenlid J., Finlay R.D., Wardle D.A., Lindahl B.D. (2013). Roots and associated fungi drive long-term carbon sequestration in boreal forest. Science.

[15] Food and Agriculture Organization (2006). Global Forest Resources Assessment 2005, Progress Towards Sustainable Forest Management.

[16] Kauppi P.E., Posch M., Pirinen P. (2014). Large impacts of climatic warming on growth of boreal forests since 1960. PLoS ONE.

[17] Bradshaw C.J.A., Warkentin I.G. (2015). Global estimates of boreal forest carbon stocks and flux. Glob. Planet. Change.

[18] Pisani O., Frey S.D., Simpson A.J., Simpson M.J. (2015). Soil warming and nitrogen deposition alter SOM composition at the molecular-level. Biogeochemistry.

[19] Startsev V.V., Mazur A.S., Dymov A.A. (2020). The Content and Composition of Organic Matter in Soils of the Subpolar Urals. Eurasian Soil Sci..

[20] Dymov A.A., Startsev V.V., Milanovsky E.Y., Valdes-Korovkin I.A., Farkhodov Y.R., Yudina A.V., Donnerhack O., Guggenberger G. (2021). Soils and SOM transformations during the two years after a low-intensity surface fire (Subpolar Ural, Russia). Geoderma.

[21] Nizamutdinov T., Bolshiianova O., Morgun E., Abakumov E. (2024). Molecular Composition of HA and SOM Stabilization Rate of the First Arctic Carbon Measurement Supersite “Seven Larches”. Sustainability.

[22] (2011). National Atlas of Soils of the Russian Federation.

[23] Kulikov A.I., Dugarov V.I., Korsunov I.M. (1997). Permafrost Soils: Ecology, Thermal Energy, and Productivity Forecast.

[24] Shahapatsev A.K., Kazeev K.S., Kozun Y.S., Soldatov V.P., Fedorenko A.N., Kolesnikov S.I. (2023). Biological activity of brown soils in old-growth clearings of the Western Caucasus. For. Bull..

[25] Kroyan S. (2018). The Contemporary State of the Humus Nutrition of the Cambisols of Republic of Armenia. Adv. Bio-Technol. Microbiol..

[26] Bayranvand M., Akbarinia M., Jouzani G., Gharechahi J., Alberti G. (2021). Dynamics of humus forms and soil characteristics along a forest altitudinal gradient in Hyrcanian forest. Biogeosciences For..

[27] Smolentsev B.A., Smolentseva E.N. (2020). Cambisols of the Kuznetsk Alatau, their properties and diversity. Tomsk State Univ. J. Biol..

[28] Zaguralskaya L.M., Morozova R.M. (2002). Microbial complexes in soils on shungites of Kizhi Island. Soil Sci..

[29] Dugarov V.I., Kulikov A.I. (1990). Agrophysical Properties of Frozen Soils.

[30] Kholodov V.A., Konstantinov A.I., Kudryavtsev A.V., Perminova I.V. (2011). Structure of HA in zonal soils from ^13^C NMR data. Eurasian Soil Sci..

[31] Grewer D.M., Lafrenière M.J., Lamoureux S.F., Simpson M.J. (2016). Redistribution of SOM by permafrost disturbance in the Canadian High Arctic. Biogeochemistry.

[32] Polyakov V.I., Abakumov E.V. (2020). Humus formation in soils of the Lena River Delta. J. Soils Environ..

[33] Semenov V.M., Tulina A.S., Semenova N.A., Ivannikova L.A. (2013). Humification and nonhumification pathways of organic matter stabilization in soil: A review. Eurasian Soil Sci..

[34] Dymov A., Zhangurov E., Hagedorn F. (2015). SOM composition along altitudinal gradients in permafrost-affected soils of the Subpolar Ural Mountains. Catena.

[35] Lodygin E., Vasilevich R. (2020). Environmental aspects of molecular composition of humic substances from soils of northeastern European Russia. Pol. Polar Res..

[36] Kaiser K., Kalbitz K. (2012). Cycling downwards—Dissolved organic matter in soils. Soil Biol. Biochem..

[37] Sollins P., Homann P.S., Caldwell B.A. (1996). Stabilization and destabilization of SOM—Mechanisms and controls. Geoderma.

[38] Egorov V.V., Ivanova E.N., Fridland V.M. (1977). Classification and Diagnostics of Soils of the USSR.

[39] IUSS Working Group WRB (2022). World Reference Base for Soil Resources.

[40] Nelson D.W., Sommers L.E., Sparks D.L., Page A.L., Helmke P.A., Loeppert R.H., Soltanpour P.N., Tabatabai M.A., Johnston C.T., Sumner M.E. (1996). Total carbon, organic carbon, and organic matter. Methods of Soil Analysis. Part 3. Chemical Methods.

[41] Shein E.V. (2001). Field and Laboratory Methods of Research of Physical Properties and Soil Regimes.

[42] Vasilevich R., Lodygin E., Abakumov E. (2018). Molecular composition of humic substances isolated from permafrost peat soils of the eastern European Arctic. Pol. Polar Res..

[43] Kovalevsky D.V., Permin A.B., Perminova I.V., Petrosyan V.S. (2000). Selection of conditions for recording quantitative 13C NMR spectra of HA. Vestn. Moscow Univ. Ser. 2 Chem..

[44] Nogina N.A. (1964). Soils of Transbaikalia.

